# Adherence to oral endocrine therapy by menopausal status: post hoc insights from a remote monitoring randomized trial

**DOI:** 10.1038/s41523-026-00900-9

**Published:** 2026-01-28

**Authors:** Ilana Graetz, Xin Hu, Rebecca A. Krukowski, Janeane N. Anderson, Edward Stepanski, Gregory Vidal, Teresa M. Waters, Lee S. Schwartzberg

**Affiliations:** 1 Rollins School of Public Health, Emory University, Atlanta, GA USA; 2 Emory University School of Medicine, Atlanta, GA USA; 3 University of Virginia Cancer Center and School of Medicine, Charlottesville, VA USA; 4 Department of Community and Population Health, College of Nursing, University of Tennessee Health Science Center, Memphis, TN USA; 5 Ovation.io, Cambridge, MA USA; 6 West Cancer Center and Research Institute, Germantown, TN USA; 7 School of Public Health, Augusta University, Augusta, GA USA; 8 Renown Institute for Cancer and University of Nevada, Reno, Reno, NV USA

**Keywords:** Breast cancer, Signs and symptoms

## Abstract

Oral adjuvant endocrine therapy (AET) improves survival in hormone receptor-positive breast cancer, but younger, premenopausal women often struggle with adherence. In a post hoc analysis of a randomized trial (*N* = 304), app-based remote symptom monitoring improved 12-month AET adherence among premenopausal women (App-only: 53.9% vs. EUC: 25.0%), with no benefit for postmenopausal women. Findings suggest remote monitoring may help close adherence gaps in younger patients. Prospectively registered on ClinicalTrials.gov: NCT03592771.

## Introduction

Despite the survival benefits of oral adjuvant endocrine therapy (AET) for women with hormone receptor-positive early-stage breast cancer, adherence rates remain low, particularly among younger, premenopausal women. Younger women have the highest risk for AET nonadherence^1,2^ and have higher risks of recurrence and breast cancer mortality ^3^, which may be partly driven by their lower AET adherence. Nonadherence among premenopausal women may stem from more pronounced side effects^4^. Tamoxifen, the standard first-line AET for most premenopausal women, frequently causes hot flashes, night sweats, and leukorrhea through its selective estrogen receptor modulation and partial estrogen agonist effects in non-breast tissues^5^. Among premenopausal women with high-risk early-stage breast cancer, tamoxifen is commonly combined with ovarian function suppression using luteinizing hormone-releasing hormone agonists, which is associated with even more abrupt and severe symptoms^6^. For postmenopausal women, who have already undergone natural estrogen decline, the additional suppression from aromatase inhibitors, the preferred AET in this group, is typically less abrupt. In addition, premenopausal women also report great quality of life disruptions from AET side effects^7^, interfering with family responsibilities, career advancement, and social interactions^8^. Many younger survivors have noted a lack of support systems designed to address their specific needs^8^. These findings underscore the need for enhanced symptom management strategies for premenopausal women prescribed AET.

Our THRIVE trial tested the effectiveness of an app-based remote monitoring intervention, with and without additional tailored text messages, on 1-year oral AET adherence, but found no significant differences between randomized groups^7^. We conducted post-hoc analyses to examine whether the effectiveness of the remote monitoring intervention varied by menopausal status, hypothesizing greater effectiveness for premenopausal women.

### Participant Characteristics

Among 304 women randomized (104 EUC, 98 App-only, and 102 App Plus Feedback), 26% (*n* = 74) were premenopausal at AET initiation. No participants had documented medication-induced menopause. Premenopausal participants were younger (mean age 45.3 vs. 63.2 years, *p* < 0.001), more likely to self-identify as Black (44.6% vs. 30.0%, *p* = 0.05), be prescribed tamoxifen (60.8% vs. 11.1%, *p* < 0.001), and have received chemotherapy (40.3% vs. 25.9%, *p* = 0.02, Table 1).

**Table 1:** Baseline characteristics by menopausal status^a^

Characteristics	Premenopausal (*N* = 74)	Postmenopausal (*N* = 230)	*P* value
Age, years			
Mean (Standard Deviation)	44.8 (6.3)	63.0 (7.8)	<0.001
Race, *n* (%)			0.05
Black	33 (44.6)	69 (30.0)	
White	38 (51.4)	154 (67.0)	
Other^b^	3 (4.1)	7 (3.0)	
Education, *n* (%)			0.38
High school or less	12 (16.2)	48 (20.9)	
Some college or more	62 (83.8)	182 (79.1)	
Income: Federal Poverty Level (FPL), *n* (%)			0.33
<100% FPL	10 (13.5)	22 (9.6)	
≥100% FPL	63 (85.1)	198 (86.1)	
Missing	1 (1.4)	10 (4.4)	
Lower health literacy^c^, *n* (%)	14 (18.9)	45 (19.6)	0.90
Married or living with a partner, *n* (%)	46 (62.2)	156 (67.8)	0.37
Location^d^, *n* (%)			0.75
Non-metro	17 (23.0)	57 (24.8)	
Metro	57 (77.0)	173 (75.2)	
Initial AET prescription, *n* (%)			<0.001
Tamoxifen	46 (62.2)	27 (11.7)	
Anastrozole	26 (35.1)	178 (77.4)	
Exemestane or Letrozole	2 (2.7)	25 (10.9)	
Cancer stage at diagnosis, *n* (%)			0.87
DCIS	8 (10.8)	26 (11.3)	
I	54 (73.0)	161 (70.0)	
II–III	12 (16.2)	43 (18.7)	
Prior chemotherapy, *n* (%)	29 (40.3)	56 (25.9)	0.02
Prior radiation, *n* (%)	47 (63.5)	141 (63.5)	1.00
Study Arm, *n* (%)			0.74
EUC	28 (37.8)	76 (33.0)	
App	23 (31.1)	75 (32.6)	
App Plus Feedback	23 (31.1)	79 (34.4)	

*DCIS* Ductal carcinoma in situ, *EUC* Enhanced Usual Care, *AET* Adjuvant Endocrine Therapy.

^a^Menopausal status was determined via chart abstraction. For the 39 participants with undocumented status, those aged <50 were categorized as premenopausal, and those aged ≥50 as postmenopausal.

^b^Among participants in the ‘Other’ race category (*N* = 10), 5 self-identified as Asian, 1 American Indian, 1 Hispanic, and 3 as mixed race.

^c^A lower health literacy is classified as responding Never/Rarely/Sometimes/Often to ‘How confident are you filling out medical forms by yourself?’, whereas a higher health literacy is classified as responding Always.

^d^Rural-Urban Commuting Area Codes (RUCA) used to categorize residential location as metro if RUCA was 1 and non-metropolitan if RUCA was 2 to 10.

### AET adherence

**Fig. 1:**
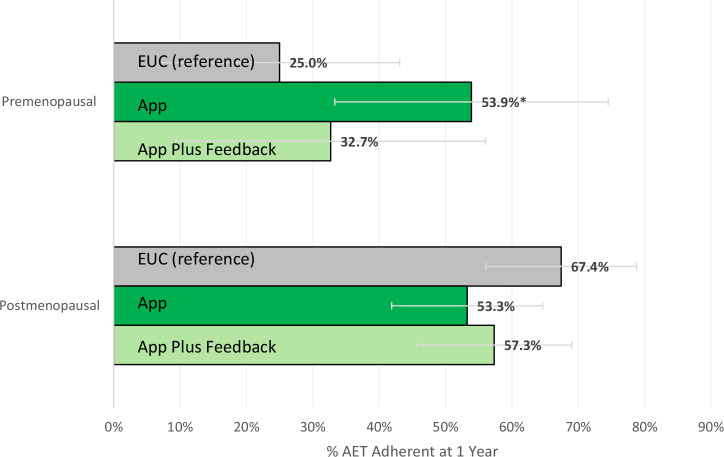
Adjusted percentage of participants AET adherent by study arm and menopausal status. One-year adherence to AET was defined as taking ≥80% of prescribed doses, as recorded by the connected pillbox. Days hospitalized and prescriber-advised pauses were excluded from the denominator. Percentages and 95% confidence intervals were estimated using a linear probability model that included an interaction term between study arm and menopausal status, followed by the margins command. Missing outcome data due to loss to follow-up (*n* = 38) were addressed using multiple imputation with chained equations (25 iterations), based on five nearest neighbors drawn from study arm, age, race, education, health literacy, marital status, rurality, income, cancer stage, prior chemotherapy/radiotherapy, and AET type. Missing menopausal status (*n* = 39) was imputed using age 50 as the cutoff based on the mean menopausal age in the US. Abbreviations: AET Adjuvant Endocrine Therapy, EUC Enhanced Usual Care, ppt percentage points. *P < 0.05.

Figure 1 shows the adjusted percentage of participants adherent to AET by study arm and menopausal status. Among premenopausal women, 25.0% in the EUC arm were adherent, compared to 53.9% in the App-only arm (adjusted risk difference [aRD]: 28.9 percentage points [ppt]; 95% CI: 1.5, 56.3) and 32.7% in the App Plus Feedback arm (aRD: 7.7 ppt; 95% CI: –21.8, 37.2).

Among postmenopausal women, there were no statistically significant differences by study arm: 67.4% in the EUC arm were adherent, compared to 53.3% in App-only (aRD: –14.1 ppt; 95% CI: –30.2, 2.0), and 57.3% in App Plus Feedback (aRD: –10.1 ppt; 95% CI: –26.6, 6.4).

The interaction coefficient showed a statistically significant 43.1 ppt greater App-only intervention effect for premenopausal versus postmenopausal women (95% CI: 11.2, 74.9), and a smaller and not statistically significant 17.8 ppt greater effect for the App Plus Feedback intervention (95% CI: –15.4, 51.0). Findings were consistent in sensitivity analyses without imputation for missing outcomes or menopausal status (aRD: 31.0 ppt for App-only vs. EUC; 95% CI: 1.9, 60.1, Supplementary Table S1).

Among premenopausal women, an app-based remote symptom and adherence monitoring intervention significantly improved 1-year oral AET adherence, but not among postmenopausal women. Consistent with prior studies^1,2^, without additional support, AET adherence in EUC was significantly lower among premenopausal women. Given their distinct adherence challenges, including higher AET-related symptom burden^2,9^, and greater comfort with technology^10^, findings from this post-hoc analysis support app-based remote monitoring as a potential scalable and effective strategy to improve adherence among younger, premenopausal breast cancer survivors. Moreover, because Black women are disproportionately diagnosed at younger ages^11^ and have lower AET adherence due to experiencing more severe symptoms^12^, targeting younger premenopausal women for remote symptom and adherence monitoring could potentially also help mitigate racial disparities in oral AET adherence.

Interestingly, among premenopausal women, participants receiving additional tailored messages showed a smaller, non-significant improvement in adherence compared to those receiving app-based monitoring alone. Prior studies found frequent messages or poorly tailored messages can sometimes lead to disengagement or message fatigue^13^. Fewer than half of App Plus Feedback participants in our study reported that the feedback messages felt personalized to their needs^14^. Nonetheless, app usage was similar across intervention arms and age groups, with over 70% of participants using the app at least half of the weeks enrolled^14^. It is possible that the additional messages offered limited benefit, or potentially reduced engagement when the messages were not perceived as personalized. These findings underscore the importance of designing feedback that is not only timely and appropriately frequent but also perceived as personally meaningful.

Among postmenopausal women, the app-based intervention, with or without additional messages, had no significant effect on 1-year adherence. This may be due to their generally higher oral adherence levels^2^, leaving less room for improvement. Additionally, older women are less likely to adopt or engage with mobile health interventions^15^. While our prior analyses found similar levels of app use across age groups, older participants were less likely to report satisfaction with the remote monitoring app^14^. Notably, AET adherence and persistence have been associated with significant disease-free survival benefits for younger, premenopausal women, with little benefit among older women^1^. Given our findings that a remote monitoring intervention was significantly more effective among premenopausal women, targeted strategies to support populations at higher risk for nonadherence may be beneficial to enhance adherence and improve survival. Conversely, postmenopausal women generally have higher AET adherence rates and may not require or benefit from additional adherence support.

Despite the study’s rigorous randomized design, several limitations should be noted. As this post-hoc analysis was not pre-specified, its findings should be confirmed in future trials focused on younger, premenopausal women. Moreover, menopausal status was imputed using age 50 as a proxy for participants with missing documented menopausal status, which may misclassify some individuals. However, sensitivity analyses excluding those with missing menopause status or primary outcome data produced similar results to the imputed findings, supporting the robustness of our findings. Adherence to other oral medications or injectables (e.g., GnRH agonists) was not captured. As a result, our adherence estimates reflect oral AET only. Additionally, our results are limited to English-speaking participants with access to a connected smartphone, all receiving care at a single large comprehensive cancer center that routinely monitored patient-reported symptoms during clinic visits. Thus, the findings may not be generalizable to other healthcare settings or broader patient populations. Future studies should aim to recruit more diverse populations, including non-English speakers, individuals without smartphone access, and treated in community-based clinics, and should capture adherence to multiple medication types, including injectables, to improve external validity.

This post-hoc analysis of a randomized controlled trial showed that remote monitoring improved oral AET adherence among premenopausal younger women, with no effect among postmenopausal women. The app combined with additional support text messages showed similar, but smaller, non-statistically significant trends for premenopausal women. These exploratory findings highlight the potential of remote therapeutic monitoring of patient reported outcomes as a scalable strategy to support oral AET adherence among premenopausal individuals, who face unique adherence challenges, are more comfortable engaging with technology, and may derive larger survival benefits from improved adherence.

## Methods

This non-blinded randomized controlled trial included women with early-stage breast cancer who were prescribed AET between November 2018 and June 2021 at a large comprehensive cancer center with 14 clinics. The enrollment survey asked about sociodemographic characteristics (race, ethnicity, educational level, household income, health literacy)^16,17^. Menopausal status and clinical characteristics were abstracted from participant’s medical charts at AET initiation. For the 39 participants with undocumented menopausal status, menopausal status was imputed using age 50 as the cutoff based on the mean menopausal age in the US^11^.

Consented participants were randomized into one of three conditions: (1) App-only, received 6 months of access to the adherence and symptom remote monitoring app, with weekly reminders, and reports of severe or increasing symptoms and missed doses triggering follow-ups from the oncology team; (2) App Plus Feedback, same as the App group plus weekly educational and motivational text messages for 6 months; or (3) Enhanced Usual Care (EUC) which included standard care with patient-reported symptom monitoring at each clinic visit. Consented patients who completed the enrollment survey were randomized via REDCap^18^ into one of three groups using race-stratified block randomization (White vs. other), with a block size of six. Stratification by race ensured balance across groups.

The THRIVE study protocol has been previously described and was approved by the University of Tennessee Health Science Center Institutional Review Board^16,17^. The study was prospectively registered on ClinicalTrials.gov (NCT03592771) on July 19, 2018.

### Outcome

The primary study outcome was 1-year AET adherence, defined as taking ≥80% of prescribed doses captured by a connected pillbox (Wisepill RT300, Wisepill Technologies). If a participant changed AET regimens during the study period, adherence was assessed across the full year, incorporating both therapies. Days during prescriber-advised pauses or hospitalization were excluded from the denominator.

### Statistical analysis

Primary analysis followed intent-to-treat principles where missing adherence data due to loss to follow-up were addressed using multiple imputations with chained equations^19^ based on five nearest neighbors drawn from study arm, age, race, education level, health literacy, marital status, rurality, income, cancer stage, prior chemotherapy/radiotherapy, and AET type conducted across 25 iterations.

A linear probability model estimated the interaction between study arm and menopausal status on 1-year adherence, using marginal effects to assess variation in intervention effectiveness by menopausal status. Sensitivity analyses were conducted without imputation for missing adherence data (*n* = 38) or participants without documented menopausal status (*n* = 39); the results are provided in Supplementary Table S1.

## Supplementary information

2025 07 14 Supplement

CONSORT_2025_editable_checklist THRIVE post hoc meno

THRIVE study protocol

## Data Availability

De-identified data used in this study are available from the corresponding author upon reasonable request.

## Code availability

The code used for these analyses is available from the corresponding author upon reasonable request.
