# Supplementary material for: Adherence to oral endocrine therapy by menopausal status: post hoc insights from a remote monitoring randomized trial
Source: NPJ Breast Cancer. 2026 Jan 28;12:33. doi: 10.1038/s41523-026-00900-9 (PMC12953915; doi:10.1038/s41523-026-00900-9)
Supplement: Supplementary file 1 — 2025 07 14 Supplement [file 41523_2026_900_MOESM1_ESM.pdf]

Table S1. Adjusted Percentage of Participants AET Adherent by Study Arm and Menopausal Status, Without Imputation (N = 236)

**Menopausal Status**	**EUC (%)**	**App (%)**	**App Plus Feedback (%)**	**App vs. EUC aRD (ppt, 95% CI)**	**App Plus Feedback vs. EUC aRD (ppt, 95% CI)**
Premenopausal (n = 60)	26.9	51.9	33.3	31.0* (1.9 to 60.1)	6.4 (-24.9 to 37.7)
Postmenopausal (n = 176)	64.5	57.9	60.0	-12.7 (-30.6 to 5.3)	-4.5 (-22.0 to 13.0)

Note: Table presents adjusted one-year AET adherence rates, defined as taking ≥80% of prescribed doses, as recorded by the connected pillbox. Hospitalized days and prescriber-advised medication pauses were excluded from the denominator.

Adjusted rates were estimated using a linear probability model with an interaction term between study arm and menopausal status. Results exclude participants lost to follow-up (n = 38) or with missing menopausal status (n = 39).

Abbreviations: AET = Adjuvant Endocrine Therapy; EUC = Enhanced Usual Care; aRD = Adjusted Risk Difference; ppt = percentage points. * $P < 0.05$.
